# Neighbourhood food and physical activity environments in England, UK: does ethnic density matter?

**DOI:** 10.1186/1479-5868-9-75

**Published:** 2012-06-18

**Authors:** Oarabile R Molaodi, Alastair H Leyland, Anne Ellaway, Ade Kearns, Seeromanie Harding

**Affiliations:** 1 MRC/CSO Social and Public Health Sciences Unit, 4 Lilybank Gardens, G12 8RZ, Glasgow, UK; 2 Department of Urban Studies, University of Glasgow, 25 Bute Gardens, G12 8RS, Glasgow, UK

**Keywords:** Obesity, Ethnicity, Neighbourhoods, Deprivation, Fast food outlets, Supermarkets, Physical activity facilities, Built environments

## Abstract

**Background:**

In England, obesity is more common in some ethnic minority groups than in Whites. This study examines the relationship between ethnic concentration and access to fast food outlets, supermarkets and physical activity facilities.

**Methods:**

Data on ethnic concentration, fast food outlets, supermarkets and physical activity facilities were obtained at the lower super output area (LSOA) (population average of 1500). Poisson multilevel modelling was used to examine the association between own ethnic concentration and facilities, adjusted for area deprivation, urbanicity, population size and clustering of LSOAs within local authority areas.

**Results:**

There was a higher proportion of ethnic minorities residing in areas classified as most deprived. Fast food outlets and supermarkets were more common and outdoor physical activity facilities were less common in most than least deprived areas. A gradient was not observed for the relationship between indoor physical activity facilities and area deprivation quintiles. In contrast to White British, increasing ethnic minority concentration was associated with increasing rates of fast food outlets. Rate ratios comparing rates of fast food outlets in high with those in low level of ethnic concentration ranged between 1.28, 95% confidence interval 1.06-1.55 (Bangladeshi) and 2.62, 1.46-4.70 (Chinese). Similar to White British, however, increasing ethnic minority concentration was associated with increasing rate of supermarkets and indoor physical activity facilities. Outdoor physical activity facilities were less likely to be in high than low ethnic concentration areas for some minority groups.

**Conclusions:**

Overall, ethnic minority concentration was associated with a mixture of both advantages and disadvantages in the provision of food outlets and physical activity facilities. These issues might contribute to ethnic differences in food choices and engagement in physical activity.

## Introduction

Obesity is a major public health concern with serious implications for the sustainability of healthcare systems globally. In the UK, the adult prevalence has risen dramatically over the last 25 years [1], with two-thirds now overweight or obese. Ethnic differences in obesity in adulthood are widely known [2]. There is also growing awareness of this disparity in childhood, particularly for Black Caribbean and Black African girls [3]. Studies have shown area of residence to be associated (independently of individual socio-economic circumstances) with obesity and physical inactivity [4-7]. Increasing exposure to obesogenic environments are thought to be a key influence on these trends as the physical and social infrastructure of neighbourhoods could discourage healthy lifestyles [8]. Neighbourhoods are assumed to affect obesity risk by influencing energy intake and energy expenditure. Some UK studies of the general population have shown that deprived areas may be less well served with physical activity facilities [9] whilst others have shown a more complex picture [10] and perceptions of unsafe or unattractive neighbourhoods may influence the likelihood of taking physical activity [11].

Studies based in the US have shown that segregation plays an important role in the distribution of neighbourhood structures. Predominantly African American neighbourhoods have more fast food outlets, fewer supermarkets and fewer recreational facilities than predominantly White neighbourhoods [12-17]. Some studies have shown a link with behaviours; African Americans consume one-third more fruits and vegetables for every additional supermarket found in a census tract [18]; food preferences appear to be partly dictated by available selection in the neighbourhoods linked to lower access to transportation in low income and African neighbourhoods [19]. In the US, segregated neighbourhoods are historically poor neighbourhoods and can be mono-ethnic [20].

The UK environment is different from that of the US in that migration to the UK from the New Commonwealth is relatively recent. It began in the 1950s, has been primarily economic (compared with forced migration of African Americans), and there are no single ethnic minority neighbourhoods [21,22]. Scotland-based studies have shown deprived areas to be better served with supermarkets and grocery stores that sell fresh fruit and vegetables [23]. Thus ethnically dense and deprived areas could be less acculturated and support traditional healthful behaviours. Vegetarianism, for example, is common in some South Asian groups and it is plausible that ethnically dense areas may be better served with fresh fruit and vegetables.

On the other hand, migration from developing to developed countries exposes ethnic minorities to more sedentary activities and energy-dense diets high in salt and fat. Lower levels of obesity and of obesity-related chronic diseases have been shown for West African populations in West Africa than in the Caribbean or the US [24]. The UK evidence on the acculturation of lifestyles is still sparse. While some studies suggest the adoption of unhealthy lifestyles [25], others suggest that the pace may differ between ethnic groups and by age and generation [3]. Ethnic minority children tend to engage more than White British adolescents in dietary practices that could promote obesity; those born abroad being less likely to engage in obesity promoting behaviours than those born in the UK [3].

In this paper we examine the association between ethnic concentration of neighbourhoods and fast food outlets, supermarkets, indoor and outdoor physical activity facilities; and whether the clustering of ethnic minorities in deprived urban areas influences the relationship. We focus on the largest ethnic groups in the England: White British, Black Africans, Black Caribbeans, Indians, Pakistanis, Bangladeshis, Chinese and Irish. Our assumption is that the supermarkets we identified are more likely to foster healthy dietary habits than fast food outlets. They have the potential to offer access to affordable, diverse foods, and generally a good supply of fruit and vegetables. There has also been progress in meeting the guidelines of the UK Food Standards Agency on reduction of salt content in own-label foods and in the provision of clear, front-of-pack labeling for fat, salt and sugar content of food [26]. However, supermarkets also sell unhealthy options and the layout of supermarkets might promote consumption (e.g. confectionaries displayed at the check out tills and fruit and vegetables at the back of the shop).

## Methods

### Neighbourhood environments

Lists of major fast food outlets (McDonald’s, Burger King, Kentucky Fried Chicken, Pizza Hut, Subway) and supermarkets (Tesco, Asda, Morrisons, Sainsbury’s) in England, were obtained from the online Yellow Pages and food outlets websites. There were 1,802 fast food outlets and 1,635 supermarkets identified in March 2009. Lists for both private and public physical activity facilities were obtained from SportEngland (http://www.sportengland.org) as of June 2009. SportEngland holds information on all public and private or commercial sports facilities in England. These were grouped as indoor (health fitness centres, ice rinks, indoor bowls, indoor tennis, sports halls and swimming pools) or outdoor (athletics tracks, golf courses, ski slopes, synthetic turf pitches and grass pitches) physical activity facilities. There were 20,461 indoor and 37, 996 outdoor physical activity facilities identified.

The full unit postcode for each fast food outlet, supermarket and physical activity facility was linked to Lower Super Output areas (LSOAs) (http://www.neighbourhood.statistics.gov.uk), which are statistical geographic units for the reporting of small area statistics in England, and the level at which area deprivation and urbanicity measures are available. There are 32,482 LSOAs (average population size of 1500, range 1000–6500) nested within 354 local authority (LA) districts. A small number of physical activity facilities could not be assigned to any LSOA as their postcodes could not be matched to those in the directory; 8 (0.04%) indoor and 208 (0.55%) outdoor physical activity facilities.

We modelled two spatial scales as it is uncertain which spatial scale might be considered a ‘walkable neighbourhood’. The first analysis was based on the facilities in the resident LSOA, and the second analysis was based on the facilities in the LSOA as well as those in contiguous LSOAs. The latter approach recognises that the facilities in an LSOA, other than the resident LSOA, may be in walking distance. We also conducted a sensitivity analysis to assess the impact of all food outlets in London rather than that of the selected large supermarkets. We used the Ordinance Survey ‘Points of Interest’ data set for London, where the density and number of ethnic minorities are highest in the UK. This data set should, in theory, cover all types of food outlets in London [27].

There are several ways to measure the distribution of ethnic groups within an area, each bearing a different potential relationship to the provision of local amenities (to the extent that preferences for particular kinds of amenities differ between ethnic groups). Ethnic composition - defined as the percentage of an LSOA's population comprised of any particular ethnic group - may influence the aggregate demand for particular goods and services within an area and thus the total number of relevant outlets. Ethnic concentration - defined as the percentage of a local authority's ethnic population (for specific ethic groups) residing in a particular LSOA - may influence the location of any ethnic-specific amenity provision within the local area in question. Thus, these measures differ in their focus on the demand or supply side of amenities, goods and services, and there is no a priori reason for preferring one measure over the other, as both are relevant to the question of amenity distribution. We present the findings based on the ethnic concentration measure. Online tables contain the findings based on the ethnic composition measure and also on a simple measure of the number of people living in each ethnic group in each LSOA. Population data from the UK 2001 census (http://www.neighbourhood.statistics.gov.uk) were used to calculate ethnic concentration within each LSOA for White British, Black African, Black Caribbean, Indian, Pakistani, Bangladeshi, Chinese and Irish.

Since for most of the ethnic groups, ethnic concentration tertile 1 (low concentration) contained LSOAs with zero representation of an ethnic group, ethnic concentration was calculated only for LSOAs where an ethnic group was present. For the White British group, this included 32,482 LSOAs (all in England), Black Africans 16,381, Black Caribbeans 19,128, Indians 24,212, Pakistanis 16,728, Bangladeshis 10,721, Chinese 21,027 and Irish 31,755. Deprivation was derived from rank of income deprivation, a domain of the Index of Multiple Deprivation (IMD 2004) and urbanicity from a three-fold Urban Rural Classification (http://www.neighbourhood.statistics.gov.uk), available for each LSOA. The three categories of Urban Rural Classification were urban, town and fringe areas and village, hamlet and isolated dwellings.

### Statistical analysis

Ethnic concentration tertiles (1 = low concentration, 3 = high concentration), and quintiles of area deprivation (1 = least deprived, 5 = most deprived) were derived. To assess the distribution of neighbourhood facilities, a widely used availability measure [28], counts of facilities relative to population [9,10,29] was used. The rates derived refer to the number of fast food outlets, supermarkets, indoor and outdoor physical activity facilities, per 10 000 population per deprivation quintile and per concentration tertile. To take account of the clustering of LSOAs within LAs, two level Poisson regression models with a random intercept were used to investigate the relationship between number of facilities and ethnic concentration. Fast food outlets, supermarkets, indoor and outdoor physical activity facilities were modelled separately with each ethnic group concentration. The baseline model was ethnic concentration specific and included the specific neighbourhood ethnic structure, adjusted for population size and clustering of LSOAs within LAs. Area deprivation was then added to these baseline models, followed by urbanicity. An interaction term of area deprivation x ethnic concentration was used to explore any differential effect of ethnic concentration. Deprivation x urbanicity interaction could not be reliably tested due to the small number of LSOAs in non-urban areas. To adjust for differences in population size in the LSOAs, an offset of the natural logarithm of the population size [30] was included in the model. The statistical analysis was carried out using MLwiN, version 2.20, using the second order penalised quasi-likelihood estimation method. To assess variance at local authority level, the median rate ratio (MRR) was obtained, calculated in the same way as the median odds ratio [31]. A value far greater 1 indicates substantial variability between local authorities.

## Results

Table 1 shows that in all LSOAs, the rates for fast food outlets and supermarkets were generally positively associated with area deprivation, higher in the more deprived areas than least deprived areas (with a tail-off for supermarkets in the most deprived areas). In contrast, the rates for outdoor physical activity facilities were higher in the least deprived areas than more deprived areas. A gradient was not observed for the relationship between indoor physical activity facilities and area deprivation quintiles, but these were less common in the most deprived areas. As Whites lived in all LSOAs, these patterns hold for the White group but not necessarily for the other ethnic groups.

**Table 1:** Mean number of facilities per 10,000 population (95% Confidence interval) by area deprivation quintile

**Facilities**	**Area Deprivation Quintile**					
	**Least deprived Q1**	**Q2**	**Q3**	**Q4**	**Most deprived Q5**	**P-value (Linearity)**
Fast food Outlets	0.17 (0.14,0.20)	0.26 (0.22,0.30)	0.33 (0.28,0.37)	0.53 (0.46,0.59)	0.57 (0.51,0.63)	0.00
Supermarkets	0.20 (0.17,0.23)	0.29 (0.25,0.32)	0.35 (0.31,0.38)	0.47 (0.43,0.52)	0.38 (0.34,0.42)	0.00
Indoor physical activity	4.18 (3.97,4.41)	4.26 (4.03,4.49)	4.31 (4.08,4.55)	4.23 (4.00,4.46)	3.86 (3.64,4.08)	0.06
Outdoor physical activity	9.69 (9.33,10.1)	9.68 (9.35,10.0)	8.27 (7.96,8.56)	6.54 (6.27,6.82)	4.36 (4.15,4.58)	0.00

Table 2 shows ethnic population sizes and ethnic concentration tertiles by area deprivation quintiles. Regardless of concentration tertile, ethnic minorities were least likely to be in the less deprived quintiles 1 and 2 than in the other deprivation quintiles. Clustering in the most deprived quintile was greatest for Bangladeshis, Pakistanis, Black African and Black Caribbeans in each concentration tertile.

**Table 2:** Percentages of ethnic population by ethnic concentration tertile and area deprivation quintile

**Ethnic concentration tertiles**^**a**^	**Own ethnic population Size (%) in LSOA**	**Area Deprivation Quintiles (Population size in ethnic specific concentration tertile = 100%)**				
		**Q1 (least deprived)**	**Q2**	**Q3**	**Q4**	**Q5 (most deprived)**
**White British**						
Low (0.01-0.72)	12472047 (29.2)	12	15	19	22	31
Moderate (0.72-1.24)	14020615 (32.8)	24	22	20	19	15
High (1.24-100)	16254474 (38.0)	26	25	23	18	8
**Black African**						
Low (0.02-0.61)	140481 (29.5)	5	9	18	35	34
Moderate (0.61-1.70)	244516 (51.4)	2	2	5	20	71
High (1.70-100)	90886 (19.1)	9	8	11	15	57
**Black Caribbean**						
Low (0.01-0.55)	158564 (28.2)	6	10	18	28	38
Moderate (0.55-1.57)	301871 (53.8)	2	3	9	29	57
High (1.57-100)	100856 (18.0)	9	9	14	21	49
**Indian**						
Low (0.05-0.50)	211798 (20.6)	10	15	19	23	33
Moderate (0.50-1.32)	386747 (37.6)	10	13	20	28	28
High (1.32-47.6)	429960 (41.8)	14	11	19	25	30
**Pakistani**						
Low (0.003-0.34)	89363 (12.6)	9	12	18	20	42
Moderate (0.34-1.35)	260935 (36.9)	4	6	11	20	60
High (1.35-100)	356276 (50.4)	3	4	9	20	64
**Bangladeshi**						
Low (0.01-0.58)	48173 (17.5)	4	6	13	20	58
Moderate (0.58-2.18)	139128 (50.5)	2	3	5	12	79
High (2.19-100)	88048 (32.0)	4	5	9	18	65
**Chinese**						
Low (0.06-0.63)	50484 (22.9)	10	13	18	23	35
Moderate (0.63-1.20)	74783 (33.9)	17	17	20	21	26
High (1.20-100)	95394 (43.2)	29	19	19	18	14
**Irish**						
Low (0.03-0.63)	197417 (31.6)	11	14	18	22	35
Moderate (0.63-1.20)	236460 (37.9)	14	14	19	26	27
High (1.20-100)	190265 (30.5)	21	21	22	22	15

^a^ Ethnic specific tertiles measured as percentage (range).

Table 3 shows the rates of provision for fast food outlets, supermarkets, indoor and outdoor physical activity facilities by ethnic concentration tertile. For the White British group, the rate of provision of fast food outlets decreased while that of indoor and outdoor physical activity facilities increased with increasing concentration. Generally for ethnic minority groups, the rates for fast food outlets, supermarkets, outdoor and indoor physical activity facilities increased with ethnic concentration. There was one exception; the rates for supermarkets did not vary by Black Caribbean concentration levels. There were two exceptions to these patterns when counts were based on resident and contiguous LSOAs (table not shown). For Black Caribbeans, the rates of fast food outlets did not vary by ethnic concentration, and for Chinese the rate of fast food outlets were highest in the moderate concentration areas. The mean number of fast food outlets /10,000 population for low, moderate and high Black Caribbean concentration areas were respectively: 5.2 (95% confidence interval 4.9-5.4); 5.6(5.3-5.8), 5.3 (5.0-5.5) and for Chinese 4.7 (4.5-5.0), 5.6(5.3-5.9), 5.0(4.8-5.2).

**Table 3:** Mean number of facilities per 10,000 population (95% Confidence Interval) by ethnic concentration tertile

**Ethnic concentration tertiles**^**a**^	**Fast food Outlets**	**Supermarkets**	**Indoor physical activity**	**Outdoor physical activity**
**White British**				
Low	0.46 (0.41,0.50)	0.33 (0.30,0.36)	3.81 (3.64,3.99)	5.37 (5.18,5.57)
Moderate	0.35 (0.32,0.39)	0.35 (0.32,0.39)	4.34 (4.16,4.52)	8.03 (7.78,8.29)
High	0.30 (0.27,0.33)	0.33 (0.30,0.36)	4.35 (4.18,4.53)	9.72 (9.47,9.98)
P-value (linearity)	0.00	0.871	0.00	0.00
**Black African**				
Low	0.41 (0.35,0.47)	0.31 (0.27,0.35)	3.72 (3.49,3.95)	4.86 (4.58,5.13)
Moderate	0.46 (0.40,0.52)	0.39 (0.34,0.43)	4.09 (3.84,4.34)	5.77 (5.45,6.08)
High	0.59 (0.52,0.66)	0.47 (0.41,0.52)	5.58 (5.29,5.88)	8.76 (8.38,9.13)
P-value (linearity)	0.00	0.00	0.00	0.00
**Black Caribbean**				
Low	0.38 (0.32,0.43)	0.34 (0.30,0.38)	3.99 (3.77,4.21)	5.54 (5.62,5.81)
Moderate	0.49 (0.43,0.55)	0.38 (0.34,0.44)	3.87 (3.65,4.10)	5.94 (5.65,6.24)
High	0.49 (0.43,0.54)	0.39 (0.35,0.44)	4.51 (4.27,4.76)	7.98 (7.66,8.29)
P-value (linearity)	0.006	0.059	0.002	0.00
**Indian**				
Low	0.37 (0.33,0.42)	0.31 (0.27,0.34)	3.68 (3.49,3.87)	5.81 (5.57,6.05)
Moderate	0.39 (0.35,0.44)	0.33 (0.30,0.36)	4.18 (3.98,4.38)	6.87 (6.59,7.14)
High	0.45 (0.40,0.50)	0.43 (0.39,0.47)	4.87 (4.65,5.10)	8.64 (8.33,8.94)
P-value (linearity)	015	0.00	0.00	0.00
**Pakistani**				
Low	0.37 (0.31,0.42)	0.29 (0.25,0.33)	3.80 (3.56,4.04)	5.84 (5.55,6.13)
Moderate	0.47 (0.41,0.53)	0.39 (0.35,0.44)	4.12 (3.88,4.36)	5.67 (5.36,5.98)
High	0.54 (0.49,0.60)	0.46 (0.41,0.51)	4.88 (4.61,5.15)	7.86 (7.51,8.21)
P-value (linearity)	0.00	0.00	0.00	0.00
**Bangladeshi**				
Low	0.45 (0.37,0.53)	0.34 (0.29,0.40)	4.04 (3.74,4.35)	4.69 (4.33,5.05)
Moderate	0.54 (0.46,0.62)	0.42 (0.36,0.48)	3.98 (3.68,4.27)	4.75 (4.41,5.09)
High	0.64 (0.55,0.73)	0.57 (0.50,0.64)	5.41 (5.05,5.78)	7.54 (7.13,7.96)
P-value (linearity)	0.003	0.00	0.00	0.00
**Chinese**				
Low	0.36 (0.32,0.41)	0.29 (0.25,0.32)	3.51 (3.31,3.70)	5.21 (4.97,5.46)
Moderate	0.43 (0.38,0.48)	0.39 (0.35,0.43)	4.25 (4.01,4.47)	6.87 (6.58,7.16)
High	0.55 (0.49,0.61)	0.52 (0.47,0.57)	5.67 (5.41,5.93)	9.36 (9.01,9.71)
P-value (linearity)	0.00	0.00	0.00	0.00
**Irish**				
Low	0.35 (0.31,0.39)	0.27 (0.25,0.30)	3.58 (3.42,3.74)	6.10 (5.89,6.32)
Moderate	0.33 (0.30,0.37)	0.33 (0.30,0.36)	4.06 (3.88,4.23)	7.47 (7.23,7.71)
High	0.44 (0.40,0.48)	0.42 (0.38,0.45)	4.93 (4.73,5.12)	9.50 (9.24,9.77)
P-value (linearity)	0.00	0.00	0.00	0.00

^a^ Ethnic specific tertiles measured as percentage (range-see Table 2).

In high ethnic concentration areas, the rates for fast food outlets, supermarkets and indoor physical activity areas were generally greater for ethnic minority groups than for the White British group. This pattern was also largely consistent for fast food outlets at moderate ethnic minority concentrations. Among the South Asian groups (Indians, Pakistanis, Bangladeshis) the rate for fast food outlets in high concentration areas was greatest for Bangladeshis. In every concentration tertile, rates for outdoor physical activity facilities were generally lower for ethnic minority groups than the White British group.

Table 4 shows rate ratios for facilities by ethnic concentration (low ethnic concentration = baseline rate), adjusted for clustering of LSOAs within local authorities, population size and area deprivation. The patterns largely correspond with those in Table 3. For ethnic minority groups, rate ratios for both fast food outlets and supermarkets increased with increasing ethnic concentration. The relative difference between low and high own concentration tertiles appeared largest for the Chinese and smallest for Black Caribbeans. For Whites, after adjustment for area deprivation, supermarkets were more likely and fast food outlets less likely, to be present in the moderate and high concentration areas than in low concentration areas. Adjusting for urban rural classification did not change these results (results not presented).

**Table 4:** **Rate ratios (95% Confidence Interval) of facilities by ethnic concentration adjusted for area deprivation**^b^

**Ethnic concentration tertiles**^**a**^	**Fast food outlets**	**Supermarkets**	**Indoor physical activity**	**Outdoor physical activity**
**White British**				
Low	1.00	1.00	1.00	1.00
Moderate	0.88 (0.77,1.00)	1.22 (1.08,1.38)	1.06 (1.00,1.12)	1.25 (1.18,1.33)
High	0.80 (0.70,0.92)	1.16 (1.02,1.32)	1.01 (0.96,1.08)	1.24 (1.16,1.32)
**Black African**				
Low	1.00	1.00	1.00	1.00
Moderate	1.04 (0.89,1.22)	1.21 (1.02,1.43)	1.13 (1.06,1.20)	1.04 (0.98,1.10)
High	1.55 (1.33,1.81)	1.51 (1.28,1.77)	1.54 (1.44,1.64)	1.18 (1.11,1.26)
**Black Caribbean**				
Low	1.00	1.00	1.00	1.00
Moderate	1.17 (1.00,1.35)	1.05 (0.90,1.23)	0.92 (0.87,0.97)	0.89 (0.85,0.93)
High	1.29 (1.10,1.51)	1.14 (0.98,1.33)	0.95 (0.88,1.02)	0.83 (0.78,0.88)
**Indian**				
Low	1.00	1.00	1.00	1.00
Moderate	1.18 (1.03,1.36)	1.14 (0.99,1.31)	1.11 (1.06,1.16)	0.97 (0.94,1.01)
High	1.54 (1.34,1.76)	1.56 (1.36,1.79)	1.29 (1.23,1.36)	0.97 (0.93,1.01)
**Pakistani**				
Low	1.00	1.00	1.00	1.00
Moderate	1.20 (1.03,1.41)	1.33 (1.13,1.58)	1.06 (1.00,1.12)	1.00 (0.95,1.05)
High	1.47 (1.26,1.72)	1.61 (1.36,1.89)	1.11 (1.05,1.18)	0.91 (0.86,0.96)
**Bangladeshi**				
Low	1.00	1.00	1.00	1.00
Moderate	1.11 (0.92,1.33)	1.21 (0.99,1.48)	0.96 (0.90,1.03)	0.89 (0.83,0.95)
High	1.28 (1.06,1.55)	1.56 (1.28,1.91)	1.08 (0.99,1.17)	0.87 (0.80,0.94)
**Chinese**				
Low	1.00	1.00	1.00	1.00
Moderate	1.46 (1.26,1.69)	1.47 (1.26,1.71)	1.27 (1.21,1.33)	1.04 (1.01,1.07)
High	2.13 (1.84,2.47)	2.05 (1.77,2.36)	1.74 (1.65,1.83)	1.09 (1.05,1.13)
**Irish**				
Low	1.00	1.00	1.00	1.00
Moderate	1.06 (0.93,1.20)	1.25 (1.10,1.42)	1.16 (1.12,1.21)	1.03 (0.99,1.07)
High	1.55 (1.37,1.76)	1.60 (1.42,1.82)	1.43 (1.37,1.50)	1.15 (1.09,1.20)

^a^ Ethnic specific tertiles measured as percentage (range-see Table 2).

^b^ Rate ratios were estimated using a two level Poisson model (LSOAs nested within LAs), with random intercepts, taking into account population size and adjusted for deprivation. Low concentration = reference.

For physical activity facilities, multilevel modelling (accounting for clustering of LSOAs within LA) changed the direction of the results shown in table 3. Table 4 shows that for indoor physical activity facilities, there was no association between density of facilities and ethnic concentration among the White British, Black Caribbean and Bangladeshis. For outdoor physical activity facilities, there was no association between density of facilities and Indian concentration, but facilities were less likely to be found in high than low ethnic concentration areas for Black Caribbeans, Pakistanis and Bangladeshis. Differences in the distribution of the facilities within LSOAs, and across LSOAs within LAs were, therefore, an important influence. For some ethnic groups variation in the outdoor/indoor physical activity facilities in the LSOAs may be due more to the level of provision throughout the LAs rather than ethnic concentration in specific LSOAs. Table 5 shows the corresponding rate ratios using a count of food outlets and PA facilities in the resident and contiguous LSOAs. The trends in rate ratios were similar to those in Table 4. The notable exceptions were for the White group and for outdoor PA. The density of supermarkets decreased, and the density of outdoor PA did not increase linearly with White concentration. For the Black African, Chinese and Irish groups, the density of outdoor physical activity decreased with increasing ethnic concentration.

**Table 5:** **Rate ratio (95% Confidence Interval) adjusted for area deprivation, using counts in resident and contiguous LSOAs**^b^

**Ethnic concentration tertile**^**a**^	**Fast food outlets**	**Supermarkets**	**Indoor physical activity**	**Outdoor physical activity**
**White British**				
Low	1.00	1.00	1.00	1.00
Moderate	0.71 (0.67,0.75)	0.96 (0.89,1.03)	0.94 (0.92,0.96)	1.12 (1.09,1.14)
High	0.49 (0.45,0.53)	0.79 (0.72,0.86)	0.78 (0.76,0.81)	0.99 (0.96,1.01)
**Black African**				
Low	1.00	1.00	1.00	1.00
Moderate	1.15 (1.09,1.21)	1.03 (0.96,1.10)	1.07 (1.05,1.09)	0.91 (0.89,0.93)
High	1.59 (1.50,1.69)	1.14 (1.05,1.24)	1.23 (1.19,1.26)	0.91 (0.89,0.93)
**Black Caribbean**				
Low	1.00	1.00	1.00	1.00
Moderate	1.06 (1.01,1.11)	0.96 (0.90,1.02)	0.95 (0.93,0.97)	0.86 (0.85,0.88)
High	1.29 (1.21,1.37)	0.99 (0.92,1.07)	1.02 (0.99,1.05)	0.79 (0.78,0.81)
**Indian**				
Low	1.00	1.00	1.00	1.00
Moderate	1.32 (1.26,1.37)	1.12 (1.06,1.18)	1.04 (1.02,1.06)	0.89 (0.88,0.91)
High	1.59 (1.52,1.66)	1.26 (1.18,1.34)	1.16 (1.14,1.18)	0.87 (0.85,0.88)
**Pakistani**				
Low	1.00	1.00	1.00	1.00
Moderate	1.21 (1.15,1.27)	1.11 (1.04,1.19)	1.03 (1.01,1.05)	0.91 (0.90,0.93)
High	1.53 (1.45,1.62)	1.29 (1.20,1.40)	1.08 (1.05,1.10)	0.84 (0.82,0.86)
**Bangladeshi**				
Low	1.00	1.00	1.00	1.00
Moderate	1.07 (1.01,1.13)	1.05 (0.97,1.13)	0.99 (0.96,1.01)	0.86 (0.84,0.89)
High	1.18 (1.10,1.26)	1.17 (1.06,1.29)	1.00 (0.97,1.04)	0.81 (0.79,0.84)
**Chinese**				
Low	1.00	1.00	1.00	1.00
Moderate	1.50 (1.44,1.57)	1.23 (1.15,1.30)	1.18 (1.16,1.20)	0.95 (0.94,0.97)
High	1.86 (1.77,1.95)	1.43 (1.34,1.54)	1.31 (1.28,1.34)	0.91 (0.89,0.92)
**Irish**				
Low	1.00	1.00	1.00	1.00
Moderate	1.29 (1.24,1.34)	1.09 (1.03,1.14)	1.10 (1.08,1.11)	0.96 (0.95,0.97)
High	1.79 (1.70,1.87)	1.29 (1.21,1.37)	1.20 (1.18,1.22)	0.94 (0.93,0.95)

^a^ Ethnic specific tertiles measured as percentage (range-see Table 2).

^b^ Rate ratios were estimated using a two level Poisson model (LSOAs nested within LAs), with random intercepts, taking into account population size and adjusted for deprivation. Low concentration = reference.

There was some area variation at local authority level for fast food outlets, indoor and outdoor physical activity facilities, ranging from 0.04, 95% confidence interval, 0.03-0.05 (MRR = 1.22) for indoor physical activity facilities among White British to 0.36, 0.29-0.42 (MRR = 1.76) for outdoor physical activity facilities among Bangladeshis. However, there was no significant area variation associated with supermarkets.

After adjusting for ethnic concentration, area deprivation remained independently and positively associated with fast food outlets and supermarkets across all ethnic groups (Additional file 1: Table S1). It was inversely associated with outdoor physical activity facilities across all ethnic groups. In contrast to the descriptive patterns in table 1, it was also independently and inversely associated with indoor physical activity facilities for all ethnic groups but Whites.

Interactions between deprivation and ethnic concentration were examined for all facilities (data not shown). Although the prevalence of fast food outlets increased with increasing deprivation among Black Caribbeans, Indians, Bangladeshis and Irish groups, there was no clear pattern by ethnic concentration in the least deprived 80%. In the most deprived quintile there were clear gradients with about twice as many fast food outlets per head of population in high concentration areas relative to low concentration areas. Similarly, in the most deprived quintile, supermarkets were more prevalent in areas with high concentration than with moderate or low concentration of Black Africans, Bangladeshis or Irish.

We conducted a sensitivity analysis to examine if the selection of specific food outlets underestimated the exposure to fast food outlets or supermarkets. Using the Points of Interest data for London, we examined the density of all fast food outlets and ‘take aways’, shops that were labeled as selling ‘Fried Chicken’, and all supermarkets, by ethnic concentration. The variation of the rates by ethnic concentration tertiles were similar to those shown in Table 3. For example, the mean number/10,000 population of fast food outlets and ‘take aways’ for low and high concentration were respectively: White British 12.01 (95% confidence interval 10.98- 13.05), 11.90 (9.98- 13.81); Black Africans 8.55 (7.65- 9.45), 13.59 (11.77-15.42); Black Caribbean 10.29 (9.12-11.46), 12.72(10.93-14.50); Indian 8.97 (8.13-9.82), 14.09(12.25-15.94); Pakistani 10.25 (9.06-11.44), 14.82 (12.74-16.89); Bangladeshi 10.49 (9.32-11.66), 15.05(12.68-17.41); Chinese 10.49 (9.32- 11.66), 15.05 (12.68- 17.41), and Irish 9.55 (8.64-10.47), 13.38 (11.51-15.25).

## Discussion and conclusions

### Principal findings

Ethnic minorities in the UK tend to live in deprived areas and our findings suggest this may not be a disadvantage in relation to some indicators of obesity promoting environments. Both fast food outlets and supermarkets were more common in deprived than less deprived areas, but outdoor physical facilities were more common in less than more deprived areas. Overall, ethnic minority concentration was associated with a mixture of both advantages and disadvantages in the provision of environments for food and physical exercise. Greater ethnic concentration was associated with more fast food outlets but also more supermarkets. Similarly, greater ethnic concentration was associated with more indoor physical activity facilities for Black Caribbeans, Pakistanis and Bangladeshis, but fewer outdoor ones. So the link between ethnic concentration and health promoting environments in these two respects was a mixed picture or double-edged sword, rather than being a simple relationship in one direction or the other. This study is the first in the UK to characterise neighbourhood environments of ethnic minorities, using objective measures of food outlets and physical activity facilities.

The findings on fast food outlets concur with those from US [12,13,15] and Canadian [32] - based studies that report a higher prevalence of fast food outlets in low than high income or in high than low ethnic minority density neighbourhoods. The findings on supermarkets concur with those of other UK-based studies for the national population [23]. Deprived areas in Scotland have been reported to have better access to grocery stores, including major supermarkets that sell fresh produce [23,33], outdoor play areas, publicly owned swimming pools, sports centres [34]. With increased exposure to supermarkets in ethnically dense areas, this might support the retention of a habit of greater consumption of fruit and vegetables among ethnic minorities than the national population in the UK [2]. As discussed earlier, however, although supermarkets can be assumed to promote healthier options than fast food outlets, they also promote unhealthy options. Compared to the national population, specific ethnic minority groups are more likely to be obese (notably adult Black Caribbean, Black African and Pakistani women), and less likely to meet recommended physical activity levels (e.g. all but the Irish among men). The findings of this study raise the possibility of amplification of these differences if the ethnic patterning of neighbourhood exposures erodes protective practices or promote habits related to obesity.

### Limitations

There are several indices of ethnic density/segregation [35].We presented ethnic concentration which gives the distribution of ethnic populations in an LA at LSOA level, on the grounds that this measure indicates the interaction with other members of own ethnic group beyond the small locality of a few streets but still within reasonable traveling distances to a facility (e.g. gyms). However, we replicated all analyses using the ethnic composition measure (Additional file 2: Table S2) and also the number of people living in each ethnic group in each LSOA (Additional file 3: Table S3). We found broadly similar results to those reported above for Tables 4 and 5. Another methodological challenge was defining the thresholds for the concentration tertiles. The distribution of ethnic concentration was highly variable across groups and the same thresholds could not be used for all ethnic groups. Ethnic specific thresholds limit formal comparisons across the ethnic groups.

Another limitation refers to temporal mismatches in the data items as ethnic density referred to 2001, area deprivation to 2004 and neighbourhood structures to 2009. This raises the question as to whether temporal changes in the composition of neighbourhoods may have shaped the availability of resources. Both ethnic dispersal and smaller ethnic cluster size have been noted in recent years rather than increasing residential segregation [36]. Measures of the neighbourhood facilities at one point in time do not reflect flux (closures/openings) in the neighbourhood structure.

It is possible that there was an underestimation of fast food exposure in the data we used. A recent qualitative study in London, found that independent outlets accommodate ethnic preferences (e.g. halal meats) in ethnically dense areas [37]. This was particularly striking in the accounts of Black Caribbean and Black African women who felt that large supermarkets did not facilitate their food choices. Other work has also suggested that small (including independent) shops may deliver generally healthy foods, better range and also more culturally appropriate foods than major supermarkets [38]. These findings correspond with those of our small field validation survey of 15 LSOAs in 4 large urban areas (London −6 LSOAs, Manchester - 3, Leicester −3, Bradford −3). We attempted to assess the completeness of the data obtained from publically available lists and found that there were more fast food outlets on the ground than in the lists, and also that the ethnically dense LSOAs had many more independent outlets than less ethnically dense areas (Molaodi et al., unpublished observations, 2011). Discrepancies between business listings and field validation findings are common [39-41].

Density does not necessary translate into usage. Some argue that access is defined by local people [38] and it is not only about the availability of food outlets but also about the quality, price and cultural appropriateness of foods/facilities [42]. Expectation of how quality and price affect choices may differ from conventional wisdom, especially in deprived areas (such as those where ethnic minorities are more concentrated). In Scotland (UK)-based studies, the quality of fresh produce was poorer in the most deprived areas [43], whilst the price of fruits and vegetables was highest in the small stores in deprived areas [44]. In addition, people may use shops or facilities available in other locations that are convenient (e.g. in/near workplace) rather than their residential place. The Scotland-based studies found that a new supermarket chain was used by people from outside the area rather the local people who continued shopping in smaller shops [45], and similarly that fast foods outlets may not be used by the locals but by those working and shopping locally or passing by [46]. We are currently examining whether neighbourhood environments affect ethnic differences in obesity and related behaviours in the UK.

Despite these limitations, a major strength of this study is that it provides insight about the potential access of ethnic minorities to neighbourhood structures that could influence their risks of obesity and more generally cardiovascular or cancer health. It lends support to a recent study which found that ethnic minority communities were concerned that the local food environment compromised their efforts to retain healthy traditional dietary habits [47]. One implication is that neighbourhood strategies to reduce barriers to maintaining health lifestyles should incorporate targeted strategies to reduce the density of fast food outlets in ethnically dense areas.

## Abbreviations

UK, United Kingdom; US, United States; LSOA, Lower Super Output Area; LA, Local Authority district; IMD, Index of Multiple Deprivation.

## Competing interests

The authors declare that they have no competing interests.

## Authors’ contributions

SH is the principal investigator and led the study. SH, AHL, AE and AK conceptualised the study, ORM linked the facilities to LSOAs, performed statistical analysis and prepared the first draft of the manuscript, and AHL gave statistical advice. All authors participated in subsequent drafts of the manuscript and approved the final version.

## Supplementary Material

Additional file 1 Table S1: Rate ratios (with 95% confidence interval) of facilities by deprivation, least deprived reference, adjusted for ethnic concentration. Click here for file

Additional file 2 Table S2: Rate ratios (with 95% confidence interval) of facilities by ethnic composition (density), least dense reference, adjusted for deprivation. Click here for file

Additional file 3 Table S3 Rate ratios (with 95% confidence interval) of facilities by ethnic population size, low population reference, adjusted for deprivation. Click here for file
